# Supplementary material for: Closed-Loop Controlled Fluid Administration Systems: A Comprehensive Scoping Review
Source: J Pers Med. 2022 Jul 18;12(7):1168. doi: 10.3390/jpm12071168 (PMC9315597; doi:10.3390/jpm12071168)
Supplement: Supplementary file 1 [file jpm-12-01168-s001.zip › jpm-1765413-supplementary.pdf]

<u>Closed-Loop Fluid administration system: A Scoping Review - Search strategy</u>

At least 1 term from each group:

<u>Group I – System</u>:

“Closed loop”

“Semi-closed loop”

“Open loop”

“Decision support”

“Automated”

“Controller”

“Adaptive control”

“Artificial Intelligence”

“Autonomous**”**

<u>Group II – purpose</u>:

“Fluid resuscitation”

“Fluid management”

“Fluid administration”

“Fluid therapy”

“Volume replacement”

Search PUBMED and SCOPUS. Limit search to publications from the last 10 years. No time limits on patent search (in SCOPUS).

<u>Search string</u>

("Closed loop" OR "semi-closed loop" OR "Open loop" OR "Decision support" OR "Automated" OR "Controller" OR "Adaptive control" OR "Artificial Intelligence" OR "Autonomous") AND ("fluid resuscitation" OR "Fluid management" OR "Fluid administration" OR "fluid therapy" OR "Volume replacement")
